# In Vitro Diagnostic Assay to Detect SARS-CoV-2-Neutralizing Antibody in Patient Sera Using Engineered ACE-2 Mini-Protein

**DOI:** 10.3390/v14122823

**Published:** 2022-12-18

**Authors:** Bruna Andersen Pereira de Jesus, Anderson Albino Gomes, Alex E. Clark, Tayse Andrade Rodrigues, Melissa Ledgerwood-Lee, Westley Van Zant, Howard Brickner, Meiqiao Wang, David L. Blum, Maria B. Cassera, Aaron F. Carlin, Eliah S. Aronoff-Spencer, Gustavo Felippe da Silva, Maria de Lourdes Borba Magalhães, Partha Ray

**Affiliations:** 1 Biochemistry Laboratory, Center of Agroveterinary Sciences, State University of Santa Catarina, Lages 88520-000, Brazil; 2 Department of Medicine, Division of Infectious Diseases and Global Public Health, University of California, San Diego, CA 92093, USA; 3 Research and Development Department, Bioclin^®^, Belo Horizonte 31565-130, Brazil; 4 Bioexpression and Fermentation Facility, University of Georgia, Athens, GA 30602, USA; 5 Department of Biochemistry and Molecular Biology and Center for Tropical and Emerging Global Diseases (CTEGD), University of Georgia, Athens, GA 30602, USA; 6 Department of Pathology, University of California, San Diego, CA 92093, USA

**Keywords:** SARS-CoV-2, spike protein, ACE2, engineered mini-protein, neutralizing antibodies, in vitro diagnostics

## Abstract

The recent development and mass administration of Severe Acute Respiratory Syndrome Coronavirus 2 (SARS-CoV-2) vaccines allowed for disease control, reducing hospitalizations and mortality. Most of these vaccines target the SARS-CoV-2 Spike (S) protein antigens, culminating with the production of neutralizing antibodies (NAbs) that disrupt the attachment of the virus to ACE2 receptors on the host cells. However, several studies demonstrated that the NAbs typically rise within a few weeks after vaccination but quickly reduce months later. Thus, multiple booster administration is recommended, leading to vaccination hesitancy in many populations. Detecting serum anti-SARS-CoV-2 NAbs can instruct patients and healthcare providers on correct booster strategies. Several in vitro diagnostics kits are available; however, their high cost impairs the mass NAbs diagnostic testing. Recently, we engineered an ACE2 mimetic that interacts with the Receptor Binding Domain (RBD) of the SARS-2 S protein. Here we present the use of this engineered mini-protein (p-deface2 mut) to develop a detection assay to measure NAbs in patient sera using a competitive ELISA assay. Serum samples from twenty-one patients were tested. Nine samples (42.8%) tested positive, and twelve (57.1%) tested negative for neutralizing sera. The data correlated with the result from the standard commercial assay that uses human ACE2 protein. This confirmed that p-deface2 mut could replace human ACE2 in ELISA assays. Using bacterially expressed p-deface2 mut protein is cost-effective and may allow mass SARS-CoV-2 NAbs detection, especially in low-income countries where economical diagnostic testing is crucial. Such information will help providers decide when a booster is required, reducing risks of reinfection and preventing the administration before it is medically necessary.

## 1. Introduction

The Severe Acute Respiratory Syndrome Coronavirus 2 (SARS-CoV-2), the causative virus responsible for the COVID-19 pandemic, has infected more than 609 million people, resulting in more than 6.5 million deaths worldwide from December 2019 to September 2022 [1]. The rapid development and mass administration of vaccines among the global population helped control the disease, diminishing the incidence of illness, and reducing hospitalizations and mortality [2,3,4]. Although this has caused a significant decline in cases since the pandemic’s peak in 2020, SARS-CoV-2 remains a severe threat to public health due to its evasion of therapeutics and diagnostics.

The outer surface of the SARS-CoV-2 virus contains the spike protein (S) that is vital in host cell attachment and viral fusion. The spike’s S1 subunit contains a receptor-binding domain (RBD) [5], which coordinates cellular attachment by binding to host angiotensin-converting enzyme 2 (ACE2) receptors. Following this binding, the spike’s S2 subunit then enables fusion with the host cell membrane [6], allowing for the genomic RNA to enter the host cell’s cytoplasm [7]. Due to its external positioning on the surface of the virion and its direct role in infectivity, the S protein is the main target for the host immune system response and neutralizing antibodies (NAbs) which target the S protein to prevent viral attachment and entry [8]. This immune response has served as the main purpose of current vaccines [9,10], developed to trigger the production of NAbs targeting several epitopes on the S protein [11,12].

Maximal neutralization of SARS-CoV-2 is achieved through a synergistic response of three immunoglobulin isotypes: IgM, IgA, and IgG [13,14]. Immediately following viral infection, IgM antibody levels rapidly increase. They primarily rise during the first week, and are usually detectable by day 4 [15]. IgM levels remain high for approximately 20 to 30 days before gradually diminishing [6]. IgA is detectable roughly 6–8 days after symptom onset [16], peaking around 20–22 days [16,17]. They rapidly decline afterward [18] yet remain detectable for up to 49–73 days post-symptoms [19]. IgG antibodies can be detected at 10–14 days following infection, [20,21,22,23] peaking at around day 25 [15]. However, IgG only has a half-life of only approximately 21 days. Therefore, while the NAb levels are seen to shoot up initially, they quickly taper off after the antigen is introduced. Of the three immunoglobulin subtypes, IgG maintains the highest levels at further time points post-infection. These sustained IgG antibody titers are likely produced by long-lived plasma cells [24].

The relatively short-lived nature of our immune response in maintaining NAbs poses serious concerns regarding the time window in which vaccine responses will remain effective. Various studies have suggested post-vaccine time points at which NAb levels may reach critically low concentrations and could no longer effectively fight infection. Some studies have indicated this to be possible 3 to 4 months after vaccination, with critically low plasma antibody concentrations allowing for infection [25,26]. Other studies have indicated that protective immunity declines after 6 or 7 months post-vaccination [27,28]. Overall, it is agreed that while levels of these molecules typically increase within the first few weeks after vaccination, they quickly taper off in the following months, reducing our ability to fend off SARS-CoV-2 infection.

Considering both the natural decrease of post-vaccine neutralizing immunity and the rapid mutation of SARS-CoV-2, evading vaccine-induced immunity, multiple booster vaccines against the virus have been recommended [8,29,30]. While many populations have adopted the recommended boosters [31], others have shown far more hesitancy [32]. Common reasons people may refuse a vaccine or booster are concerns about the safety or efficacy of the vaccine, a general lack of fear of the disease, and the belief to be already immunized by a previous infection or vaccine [33]. This is compounded by multiple booster recommendations, as people question their prior vaccination’s efficacy. Further complications arise when considering the longevity of the protection provided by vaccines. Variations in people’s immune responses following vaccination could cause some to require re-vaccination sooner than others.

On the other hand, wide-scale redundant vaccinations can also have adverse side effects and place unnecessary burdens on the healthcare system. Therefore, further efforts should be made to provide people with the necessary information about vaccines and their current immunity levels. This can prevent high vaccine hesitancy affecting individual health and public safety.

In vitro diagnostics kits are available to determine the presence of SARS-CoV-2 NAbs in patient sera. One such kit is based on a competitive Enzyme-Linked Immunosorbent Assay (ELISA) (Bioclin^®^ ref. K243). Briefly, the ACE2 protein immobilized on the micro-well plates binds to the HRP-conjugated S protein and produces a colorimetric signal in the presence of the enzyme’s substrate. However, NAbs present in the patient’s sera cause a competition reaction between ACE2 and NAbs for binding to the S protein, which decreases its availability to binding ACE2. This results in a concomitant decrease in the ELISA colorimetric signal. Conversely, when NAbs levels are low, the S protein can bind the ACE2 without competition, which results in a high signal. This result can alert the patient or practitioner that it is time for revaccination. However, these commercial kits use ACE2 proteins, which makes them far too expensive for equitable distribution and repeated mass testing.

In our previous publication, we engineered a short alpha-helical segment of the ACE2 protein that interacts with the Receptor Binding Domain (RBD) of the SARS-2 S protein on a defensin scaffold [34]. The engineered mini-proteins were produced in a bacterial expression system with a high yield and demonstrated exceptional thermostability and high affinity binding to the S protein. These peptides mimic the ACE2 receptor and can bind the S protein while being more viable to produce at scale.

Here we present the use of the engineered mini-protein to develop an in vitro diagnostic assay that can be used to indicate the presence of NAbs in patient sera and, consequently, when a COVID-19 booster is warranted. The assay is based on the competitive ELISA, where we replaced the ACE2 protein in the Bioclin kit with our engineered mini-protein to determine the patient’s NAbs. Since the assay uses the bacterially produced ACE2 mimetic mini-protein, the price associated with the production of full-length ACE2 can be minimized. Therefore, we anticipate that the overall cost of the kit would be affordable for mass testing. This information will help healthcare providers and patients make more informed decisions about when to boost their vaccine regimen. Providing information on when a booster is required reduces risks of reinfection by eliminating the threat of providing the vaccine too late. Conversely, it would help in preventing the administration of a booster before it is medically necessary. This could improve the overall efficiency and confidence of our healthcare system by providing direct information on when a booster is vital.

## 2. Material and Methods

### 2.1. Protein Expression and Purification

Protein expression and purification followed the earlier protocol [34]. Briefly, recombinant pET-32a (+)::p-deface2-mut was transformed into *Escherichia coli* BL21(DE3) pLysS and plated on Luria-Bertani (LB) agar plates containing the antibiotics ampicillin (100 μg/mL) and chloramphenicol (37.5 μg/mL). Isolated colonies were selected and inoculated into 1-L LB expression media containing the same antibiotics and incubated at 37 °C until OD_600 nm_ reached 0.6 in absorbance. IPTG (0.1 mM) was added, and cells were induced at 37 °C for 7 h. Cells were collected by centrifugation at 1000× *g* for 10 min and suspended in 20 mL Buffer A (50 mM Tris-HCl, pH 8.0, 500 mM NaCl) containing 1 mM phenylmethylsulfonyl fluoride (PMSF) and lysozyme (10 μg/mL). Cells were sonicated on the ice, debris was removed by centrifugation at 10,000× *g* for 10 min, and the supernatant was applied into a Ni-NTA Sepharose. The resin was washed with buffer B (Tris-HCl 50 mM pH 8.0; 500 mM NaCl; 60 mM imidazole) to remove weakly bound proteins, and p-deface2-mut mini protein was eluted in buffer C (500 mM NaCl, 500 mM Imidazole; 50 mM Tris-HCl, pH 8.0).

### 2.2. SARS-CoV-2-Neutralizing Antibody ELISA

Bioclin^®^, Belo Horizonte, Brazil, provided all the serum samples; the company receives human sera from a local hospital laboratory as a donation for internal test validation.

The presence of neutralizing antibodies in serum samples from 21 patients was previously tested by the company Research and Development team using the Biolisa COVID-19 neutralizing antibody kit (Bioclin^®^ ref. K243). The kit was approved by the Brazilian National Sanitary Agency (ANVISA).

The Biolisa COVID-19 neutralizing antibody kit is a competitive solid-phase immunoassay that detects anti-SARS-CoV-2 antibodies capable of inhibiting the Spike-ACE2 complex. According to the manufacturer´s information, the sensitivity (96.8%) and specificity (95%) were initially determined using 156 samples previously tested for the presence of SARS-CoV-2-neutralizing antibodies. Furthermore, a new clinical sensitivity study was carried out with 70 positive samples, tested using the Plaque Reduction Neutralization Test -PRNT gold-standard method, and 100 negative (pre-pandemic) samples. This new study yielded a sensitivity of 97.1% and a specificity of 92.0%.

The assay is based on a competitive ELISA where biotinylated ACE2 is initially captured on streptavidin-coated plates, followed by incubation with HRP-coupled Spike protein in the presence of serum samples. The binding of HRP-Spike protein to the immobilized ACE2 generates a colorimetric signal in the presence of HRP substrate. However, in the presence of anti-SARS-CoV-2-neutralizing sera, the availability of the Spike protein to ACE2 binding decreases, resulting in a reduction of the measured signal.

Twenty-one human serum samples provided by the company were subjected to a blinded study using p-deface2-mut to replace human ACE2 on ELISA assay. Results from the modified ELISA assay were compared to results obtained using the ANVISA-approved kit.

For the assay, 1 µg of p-deface2-mut mini protein was diluted into 100 µL PBS buffer (pH 7.4) and coated directly onto a 96-well high-affinity polystyrene plate for 3 h at 37 °C. Plate wells were washed five times with 200 µL PBS-T (PBS pH 7.4, 0.05% Tween-20). The plate was blocked with 200 µL BSA (1%) in PBS (pH 7.4) overnight at 4 °C and washed with PBS-T.

For our analysis, each serum sample was diluted (1:10) in PBS, and all the following steps were performed according to the original protocol from the manufacturer. Ten microliters (10 µL) of diluted serum samples were added to the wells, followed by the addition of 50 µL of BIOLISA kit diluent and 100 μL of HRP-Spike protein (Bioclin^®^ kit-K243). Wells were mixed, covered with a plate sealer, and incubated for 30 min at 37 °C, followed by five washing steps with PBST. The reaction was visualized by adding 100 μL chromogenic substrate TMB for 20 min and quenched with the addition of 100 μL stop solution, and the absorbance at 450 nm was measured using an ELISA plate reader.

### 2.3. Calculation and Interpretation of the Results

Serum samples used in this study were previously analyzed using the ANVISA-approved kit (Bioclin^®^ kit-K243) to classify samples as true negative or true positive regarding the presence of neutralizing antibodies. According to the manufacturer´s protocol, the percentage of neutralization can be calculated as shown below (Equation (1)).

(1) $$\%\;of\;neutralization=\left[1-\left(\frac{sample\;abs}{negative\;control\;abs}\right)\right]\times \;100\;$$

where *sample abs* are the absorbance at 450 nm of the tested sample and *negative control abs* are the absorbance at 450 nm of the negative control provided with the kit.

Results are presented as a percentage of inhibition, where samples showing <30% inhibition do not contain neutralizing antibodies, samples resulting in >35% neutralization have SARS-CoV-2-neutralizing antibodies, and percentages between 30 and 35% are considered undetermined.

When the p-deface2-mut mini protein was used in the assay to replace immobilized ACE2, the percentage of neutralization was also calculated following Equation (1), except that negative control absorbance was determined using a true negative serum sample (Table 1, Sample ID# 1) previously analyzed from the company’s serum bank.

We also determined the positive percent agreement and negative percent agreement to determine the sensitivity and specificity of the modified test, respectively, in agreement with the ANVISA-approved test Bioclin^®^ kit-K243.

Sensitivity (*S*) and Specificity (*Sp*) were calculated according to Equations (2) and (3), below.

(2) $$S=\left[\frac{TP}{TP+FN}\right]\times 100$$

(3) $$Sp=\left[\frac{TP}{TN+FP}\right]\times 100$$

where *TP* is the number of truly positive samples, *TN* is the number of truly negative samples, *FP* is the number of false-positive samples, and *FN* is the number of false-negative samples.

### 2.4. Detection of SARS-CoV-2 Spike Protein Variants

ELISA assays were performed in 96-well high binding microtiter plates. Wells were coated (18 h, 4 °C) with 10/25/50 ng of Spike RBD variants Wuhan-Hu-1 (NR-52307), Omicron-B.1.1.529 BA.2 lineage (NR-56517), Alpha (NR-55277) or Beta (NR-55278) or Bovine Serum Albumin in 50 μL PBS (Phosphate buffer saline) and blocked with 200 mL of 1% BSA in TBST (PBS, 0.05% Tween-20) for 2 h at 37 °C. The RBD variants were obtained through BEI Resources, NIAID, and NIH. Next, the biotinylated mini protein was diluted in TBST, 1% BSA, and added to the wells to a final volume of 100 μL. Finally, one hundred microliters of High Sensitivity streptavidin-HRP (Thermo Scientific ^TM^ Pierce ^TM^, Waltham, MA, USA) (dilution 1:400) was added to the wells and incubated for 15 min at room temperature. Plates were washed five times with PBST after each step, except for the final washing, which included eight washing steps. The reaction was visualized by adding 50 μL Ultra TMB-ELISA Substrate Solution (Thermo Scientific ^TM^, Waltham, USA) for 15 min. The reaction was quenched with 50 μL of 0.16 N sulfuric acid, and absorbance at 450 nm was measured using an ELISA plate reader.

### 2.5. Cell Toxicity Analysis

293T cells (7500 cells per well in 100 μL media) were plated in 96-well plates and treated with the respective proteins at 0.1 and 0.2 μg/μL (final concentration). Each application was performed in triplicate. The cells were incubated under normal tissue culture conditions for 24 and 48 h. Following this, the cells were assayed for cell viability by using AquaBluer Fluorescent Redox indicator (MultiTarget Pharmaceuticals LLC, Colorado Springs, CO, USA. Catalogue-6015) reagent following the manufacturer’s protocol.

The percentage of cell viability was calculated according to Equation (4):

(4) $$\%\;Cell\;viability=\left[\frac{Fluorescence\;intensity\;of\;tested\;cells}{Fluorescence\;intensity\;of\;cells\;in\;vehicle}\right]\times 100$$

### 2.6. Neutralization Assay

Authentic SARS-CoV-2 isolate WA1 (USA-WA1/2020, BEI NR-52281) was propagated on TMPRSS2-VeroE6 cells (XenoTech, Kansas City, MO, USA) and tittered by fluorescent focus assay on TMPRSS2-VeroE6 cells. Viral stocks were verified by whole genome sequencing. Focus reduction neutralization test (FRNT) was performed as previously described [35] with modifications. TMPRSS2-VeroE6 cells were plated in 96-well plates (12,000 cells per well in 100 µL media) one day before infection. Ten-fold serial dilutions of mini proteins or anti-spike antibody control (Sino Biological, Beijing, China, 40592-MM57) were made in DMEM plus 1% FBS. Dilutions were incubated with 150 focus forming units (FFU) of SARS-CoV-2 diluted in DMEM in a total volume of 30 µL in round-bottom 96-well plates for 1 h at 37 °C, 5% CO_2_. Cells were washed once with DPBS, then 25 µL of virus + mini protein mixture was transferred to cells and incubated for 1 h at 37°, 5% CO_2_ with gentle rocking. The virus was removed, and wells were overlaid with 1% methylcellulose in MEM with 2% FBS and 1× penicillin/streptomycin. After incubation for 24 h, overlays were removed, and cells were fixed with 4% formaldehyde for 30 min and stained with anti-nucleocapsid primary antibody (GeneTex, Irvine, CA, USA, gtx135357) and anti-rabbit AlexaFluor 594 secondary (Thermo Fisher Scientific, Waltham, MA, USA) with Sytox Green nuclear counterstain. Whole well images were acquired on an Incucyte S3 (Sartorius), and foci were counted using the Incucyte onboard software tools. The assay was run in triplicate. Best fit curves for µg/mL vs. #foci were created in GraphPad Prism 9 using a variable slope nonlinear curve fit model with top unconstrained and bottom = 0. All work with SARS-CoV-2 was conducted in Biosafety Level-3 conditions at the University of California San Diego, following the guidelines approved by the Institutional Biosafety Committee.

## 3. Results

### 3.1. SARS-CoV-2-Neutralizing Antibody ELISA

Serum samples from twenty-one patients were analyzed in this study. Samples were previously tested using Bioclin kit-K243 to detect SARS-CoV-2-neutralizing antibodies. Nine samples (42.8%) were tested as positive, and twelve samples (57.1%) were tested as negative for the presence of NAbs according to the ANVISA-approved Bioclin kit-K243 (Table 1).

We modified the assay and replaced the biotinylated-ACE2 from the original kit with the ACE2 mimetic developed by our group (p-deface2-mut). The mini protein was directly coated onto a polystyrene plate, circumventing the need for biotinylation and capture into streptavidin-coated plates. This modification removed one step from the original protocol, reducing the total time of analysis by 40% and production costs.

**Figure 1:**
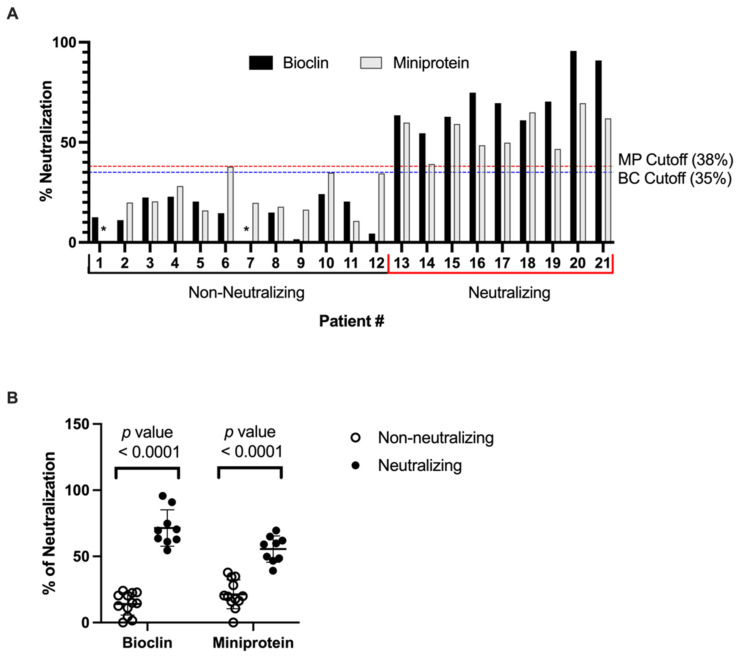
Detection of SARS-CoV-2-neutralizing antibodies from patients’ serum samples. (**A**) The percentage of neutralization for each tested sample was calculated based on Equation (1), except that negative control absorbance was determined using the most negative serum sample. For the mini protein assay, sample 1 (indicated by *) was considered a negative control for the calculation, while sample 7 (indicated by *) was considered a negative control for the commercial kit. For commercial kits, according to the manufacturer’s protocol, sera presenting neutralization >35% was considered positive to the presence of neutralizing antibodies. For the mini protein-modified assay, a 38% cutoff was determined to discriminate between positive and negative samples. (**B**) Both the commercial (Bioclin, BC) assay, using human ACE2, and the Miniprotein, MP (p-deface2 mut) assay discriminated the SARS-CoV-2-Neutralizing (*n* = 9) vs. Non-neutralizing (*n* = 12) sera with high statistical significance (Mann-Whitney, *p*-value <0.0001). Bioclin Kit, Neutralizing vs. Non-neutralizing sera (Mean: 71.5 vs. 14.1). Miniprotein (p-deface2 mut) Kit Neutralizing vs. Non-neutralizing sera (Mean: 55.5 vs. 21.4).

Data analysis demonstrates that p-deface2 efficiently captured HRP-coupled Spike protein and demonstrated that the presence of neutralizing sera disrupted this interaction, thus decreasing the measured signal compared to non-neutralizing sera (Table 1). Graphs presenting the percentage of neutralization of tested sera using both assays are shown in Figure 1. A cutoff of 38% was proposed to discriminate positive and negative samples, resulting in the successful identification of all nine positive neutralizing sera using the modified assay and confirming that p-deface2-mut can be directly applied into an ELISA-based diagnostic assay platform to screen SARS-CoV-2-neutralizing sera.

A cutoff of 38% neutralization was chosen to discriminate neutralizing from non-neutralizing sera, resulting in the absence of false positive or false negative samples, therefore, resulting in 100% Sensitivity and Specificity of the modified assay (Table 2).

This study validates the use of the engineered mini protein as an economical reagent for NAbs detection in the ELISA platform, possibly also useful in additional NAbs detection methods such as LFIAs or biosensors. The developed mini protein represents a promising tool for neutralizing antibody detection as well for the screening of small molecule libraries that can disrupt the binding of ACE2 and the viral Spike protein.

**Figure 2:**
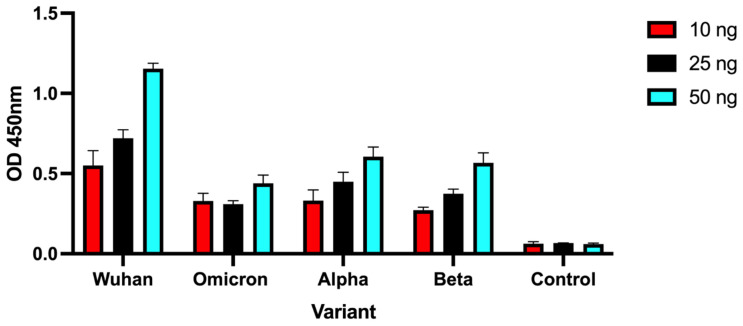
Detection of spike protein variants using engineered mini protein. Different quantities of spike RBD variants or Bovine Serum Albumin (control) were immobilized into wells and allowed to bind biotinylated mini protein. Detection was achieved by the addition of streptavidin-HRP and peroxidase substrate. Bars represent the average OD measurements performed in duplicates, and error bars represent standard deviation.

We also investigated the ability of engineered mini-protein to bind Wuhan-Hu-1, Omicron, Alpha and Beta spike variants using standard ELISA assays. Figure 2 shows OD _450nm_ reading using 10, 25 or 50 ng immobilized RBD variants, demonstrating that although the Wuhan signal is higher, the mini protein was also capable of binding to the tested SARS-CoV-2 variants.

### 3.2. Neutralization Assay

**Figure 3:**
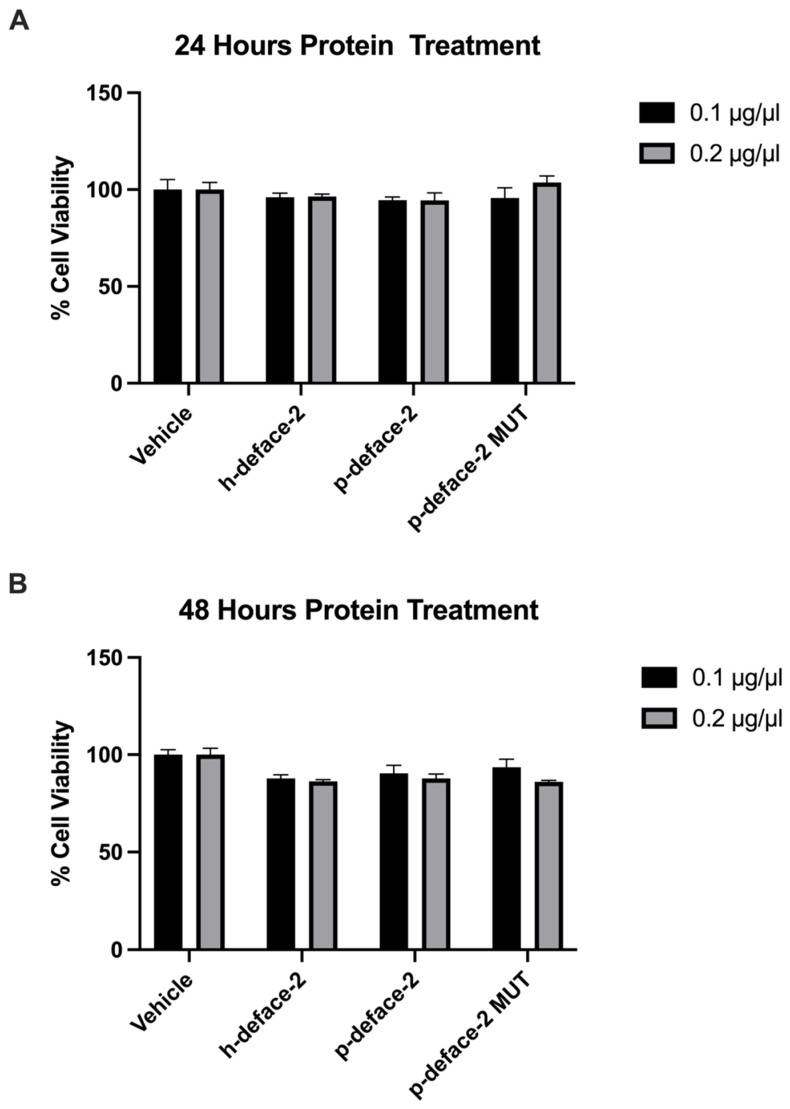
Cell viability assay. 293T cells were treated with h-defaec2, p-deface2, and p-deface2 MUT mini-proteins, and the cell viability was assayed after 24 and 48 h. The percentage of cell viability, which is defined as the percentage fluorescence of protein-treated cells compared to the fluorescence of vehicle-treated cells, is plotted. Experiments were performed in triplicate (*n* = 3).

Next, we wanted to test if the engineered mini-proteins can neutralize SARS-CoV-2 infection. Before that, we tested the effect of the proteins on cell viability. We incubated the mini-proteins at 0.1 and 0.2 µg/µL with 293T cells for 24 and 48 h and performed a cell viability assay. Compared to the control, the engineered proteins demonstrated no cellular toxicity at the 24 h time point (Figure 3A) and a very modest cell-inhibition effect at 48 h (Figure 3B).

**Figure 4:**
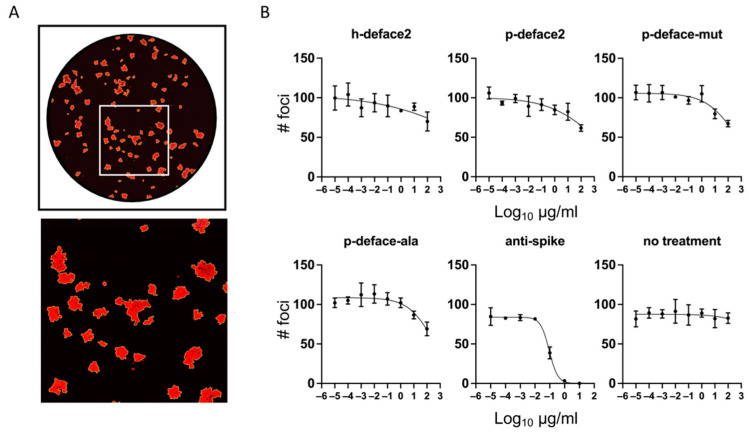
Mini-proteins do not neutralize infection of authentic SARS-CoV-2. Focus reduction neutralization test (FRNT) of SARS-CoV-2 (WA1 strain) on TMPRSS2-VeroE6 cells. Ten-fold dilutions of mini-proteins, alanine mutant negative control, anti-spike antibody positive control, or media alone were incubated with authentic SARS-CoV-2 and used to infect cells as described in Methods. (**A**) Representative whole-well image (above) with a magnified portion (below) indicated by the square. (**B**) Graphs and best-fit curves of the number of foci counted by Incucyte S3 software. Graphs show the mean and SD of the assay run in triplicate. NT50 and SEM of best-fit curves are displayed in Table 3.

Following this, we tested the neutralization assay using a focus reduction neutralization test (FRNT) with authentic SARS-CoV-2 (USA-WA1/2020) on TMPRSS2-expressingVeroE6 cells. Serial dilutions of mini-proteins were incubated with the virus in triplicate. The mixtures were applied to cells for 1 h, then replaced with a viscous overlay for 24 h, and foci of infection were visualized by immunofluorescence against Nucleocapsid protein (Figure 4A). Anti-spike neutralizing antibody was used as a positive control, and triplicate untreated rows (no treatment) indicate any plate effects. Best fit curves (Figure 4B) and half maximal virus neutralizing concentrations (NT50) (Table 3) were calculated from counts of foci taken from whole-well images.

All mini-proteins tested showed some reduction in the number of foci at the highest concentration tested (100 µg/mL). The reductions did not reach the extrapolated NT50 and were also present in the alanine mutant, indicating a non-specific effect on the infection (Figure 4B, Table 3). Control anti-spike antibody neutralized infection with an NT50 of 91 ± 9 ng/mL. This suggests that the mini-proteins bind the spike protein by a mechanism that does not exclude infection.

## 4. Discussion

The replacement of biotinylated-ACE2 with the engineered p-deface2-mut mini-protein resulted in a functionally analogous assay that successfully detected the presence of neutralizing antibodies against the SARS-CoV-2 spike protein. Just as the original test kits exhibited a decreased signal resulting from the disruption in the interaction between biotinylated-ACE-2 and HRP-conjugated SARS-CoV-2 spike protein, a similar signal decrease resulted from the disruption of the interaction between the HRP-conjugated spike protein and our modified peptide in the presence of anti-SARS-CoV-2 NAbs. The similar signal decrease observed indicates the ability of the engineered peptide to functionally serve in neutralizing antibody tests. In addition, the mini-protein-based assay successfully detected all nine positive neutralizing sera, exhibiting similar signal decreases compared to the original test kit. The comparable functionality between this modified assay and the original test kits indicates the viability of our mini-protein as an alternative to the actual ACE2 protein in these diagnostics. Additionally, results demonstrated that mini-protein was capable of binding all four SARS-CoV-2 spike variants, being a promising molecular tool for Nabs detection against clinically important variants. Protection against COVID-19 also involves innate and T-cell responses [36]; however, our assay will not be suitable to measure them.

While there were initial hopes of broadening the applications of the mini-protein to therapeutic applications in addition to its diagnostic uses, it could not neutralize SARS-CoV-2 infection. While binding was possible, it was not conducive to neutralization. The defensin scaffold has positively charged amino-acid residues, and the charge-charge interactions may cause the mini-proteins to bind to the negatively charged phospholipids of the cellular membrane. This general attraction between the defensin-based engineered mini-proteins, and cellular membranes could explain the almost equal neutralizing activity between the defensin-ACE-2 mini proteins and the alanine mutant at the peptide concentrations that were used. This does not, however, eliminate the possible viability of defensin-based mini-proteins in therapeutic applications. Future changes to the charged amino acids in the defensin backbone of the engineered mini-protein could eliminate unwanted binding interactions.

Peptides such as these are particularly beneficial in improving the scalability and affordability of mass-market diagnostics. The critical financial constraint of existing NAb test kits is the use of full-length ACE2 proteins since their difficult and cumbersome production imposes limitations on the viability of the wide-scale use of these kits. Their high price makes them unattainable in low-resource settings, and with cheaper alternatives, the critical information they provide is attainable in areas most impacted by COVID-19. Because these ACE2 mimics are designed to function similarly to the actual protein used in existing diagnostics, they can serve as alternatives to the protein. Replacing the ACE2 proteins in existing test kits with functionally similar engineered mini-proteins allows the tests to still functionally detect the presence of NAbs without relying on the expensive ACE2 proteins. The simpler and quicker production required of these alternative mini-proteins allows the kits to be produced in greater volumes. This can lower the overall price of diagnostic kits, allowing them to be used in more economically diverse settings and utilized by a more significant portion of the overall population. Because defensin-based mini-proteins like p-deface2-mut are significantly stable, they will offer longer-term shelf life than products utilizing the natural ACE2 protein. Mini-proteins can provide a promising alternative to the ACE2 protein in neutralizing antibody tests, offering functional parity to the natural protein while eliminating cost, production, and scalability constraints that limit current testing kits. With the tremendous structural control offered by mini-proteins, similar rigidified helices could see future uses in diagnosing and treating other diseases, both in modifying existing practices and in novel therapeutic and diagnostic methods.

## 5. Conclusions

We explored using an ACE-2-based mini-protein to replace the ACE-2 receptor in a commercial COVID-19-neutralizing antibody test kit. Retaining the ability to detect spike-neutralizing sera, the modified assay indicated its viability as a functional alternative to existing diagnostics. The assay, however, is not yet optimal; while it behaves on par with the original commercial kit, it requires further optimization and testing with a larger sample size. Nonetheless, assays such as these could provide a cost-effective alternative for a qualitative assay to determine the presence or absence of neutralizing sera, both for patients who have received anti-Spike protein vaccines and those who have recovered from COVID-19.

## Figures and Tables

**Table 1:** Neutralizing antibody ELISA assay.

		Bioclin Assay ^1^		Mini Protein Assay ^2^			
Sample	Result	Abs (450 nm)	Neutralization (%)	Abs (450 nm)	Abs (450 nm)	Abs Mean	Neutralization (%)
1	Negative	2.141	5.47	0.535	0.509	0.522	0
2	Negative	2.178	3.84	0.427	0.409	0.418	19.9
3	Negative	1.901	16.07	0.506	0.324	0.415	20.4
4	Negative	1.892	5.40	0.45	0.30	0.375	28.1
5	Negative	1.950	6.91	0.465	0.412	0.439	15.9
6	Negative	2.093	7.59	0.38	0.269	0.325	37.8
7	Negative	2.450	8.16	0.462	0.376	0.419	19.7
8	Negative	2.086	14.36	0.448	0.41	0.429	17.8
9	Negative	2.412	6.49	0.396	0.477	0.437	16.3
10	Negative	1.859	7.05	0.365	0.316	0.341	34.7
11	Negative	1.949	13.95	0.507	0.425	0.466	10.7
12	Negative	2.342	3.40	0.349	0.335	0.342	34.4
13	Positive	0.894	55.65	0.233	0.186	0.210	59.8
14	Positive	1.114	41.37	0.339	0.296	0.318	39.1
15	Positive	0.912	54.76	0.245	0.182	0.214	59.0
16	Positive	0.617	66.17	0.277	0.26	0.269	48.5
17	Positive	0.745	63.05	0.266	0.258	0.262	49.8
18	Positive	0.956	47.59	0.193	0.173	0.183	64.9
19	Positive	0.725	64.04	0.27	0.287	0.279	46.6
20	Positive	0.106	94.74	0.153	0.165	0.159	69.5
21	Positive	0.224	88.21	0.18	0.217	0.199	61.9

Acquired data from ELISA assays using the Bioclin kit (^1^) or the modified mini protein ELISA assay (^2^). Mini protein assay was performed in duplicates, and the absorbance means is also presented. Sample #1 was used as a negative control for calculating % neutralization using the modified assay. Negative samples are highlighted in gray.

**Table 2:** Parameters found for the ELISA modified NAb detection assay.

Number of total tested samples	21
Number of truly negative samples	12
Number of truly positive samples	9
Number of false-positives	0
Number of false-negative	0
Cut-off	38%
Sensitivity	100%
Specificity	100%

**Table 3:** Half maximal virus neutralizing concentrations (NT50) and SEM from best-fit curves from focus reduction neutralization test (FRNT) of authentic SARS-CoV-2 described in Figure 4.

	NT50 (µg/mL)	SEM
h-deface2	8.9 × 10^4^	4.7 × 10^5^
p-deface2	1.2 × 10^3^	1.5 × 10^3^
p-deface-mut	3.8 × 10^2^	2.6 × 10^2^
p-deface-ala	3.1 × 10^2^	1.9 × 10^2^
anti-spike	9.1 × 10^−2^	8.8 × 10^−3^
no treatment	1.1 × 10^5^	2.0 × 10^6^
